# Ammonium ion concentration-dependent production of extracellular polysaccharides from glycerol by an oleaginous yeast: visualization of the producer cells

**DOI:** 10.1128/spectrum.00429-26

**Published:** 2026-06-29

**Authors:** Soyoka Takegawa, Chiaki Matsuzaki, Taiki Hikosaka, Yuji Okabe, Koji Kawai, Shigeyuki Kawai

**Affiliations:** 1 Department of Environmental Science, Faculty of Bioresources and Environmental Sciences, Ishikawa Prefectural University 73985, Nonoichi, Ishikawa, Japan; 2 Laboratory for Environmental Biotechnology, Research Institute for Bioresources and Biotechnology, Ishikawa Prefectural University 73985, Nonoichi, Ishikawa, Japan; 3 Miyoshi Oil & Fat Co., Ltd., Tokyo, Japan; University of Maryland Baltimore County, Baltimore, Maryland, USA

**Keywords:** *Rhodotorula toruloides*, extracellular polysaccharide, crude glycerol, oleochemical industrial waste, lipid droplet

## Abstract

In the oleochemical industry, a fat-splitting process is used to generate a methanol-free byproduct, oleochemical industrial waste, that includes crude glycerol. The oleaginous yeast *Rhodotorula toruloides* is a nonconventional yeast that is well known for its industrial potential as a producer of lipids, carotenoids, and enzymes. Extracellular polysaccharides (EPSs) are of increasing interest due to their many applications. In this study, we found that *R. toruloides* NBRC 8766 strain produced mannose-rich and high-molecular-weight EPSs with a mannose content (mol%) exceeding 90% and an estimated molecular weight greater than 10,000 k from oleochemical industrial waste crude glycerol or pure glycerol. Components in the industrial waste increased EPS production. The concentration of NH_4_^+^ ions in the culture medium was identified as a key factor in discriminating the cells producing the EPS (EPS-producing cells) from EPS-nonproducing cells. Further investigations demonstrated that a sufficient concentration of NH_4_^+^ ions (38 mM or more) to acidify the medium to a pH of 2.0 or lower is key to EPS production under these conditions. Therefore, it was hypothesized that the *R. toruloides* NBRC 8766 strain would produce EPS in response to extracellular acidity. Fluorescent and transmission electron microscopic analyses of the two cell types revealed differences; the EPS-producing cells had immature lipid droplets and marked two-layered cell walls, where the outer layer was darker and looked like it was wearing “fuzz,” which was assumed to be the EPSs released from the cells. The EPS-nonproducing cells had clear lipid droplets, one-layered cell walls, and more abundant intracellular polysaccharide granules.

**IMPORTANCE:**

In this study, we demonstrated that oleochemical industrial waste is a promising source of crude glycerol for EPS production and that *R. toruloides* is a promising producer of not only lipids but also EPSs from glycerol. Moreover, the identification of the concentration of NH_4_^+^ ions in the culture medium enabled the discrimination of EPS-producing cells from EPS-nonproducing cells. The presence of sufficient NH_4_^+^ ions to acidify the medium was demonstrated to be key to EPS production in these conditions. This suggests that the *R. toruloides* NBRC 8766 strain produces EPS in response to extracellular acidity. The morphological observations of the EPS-producing and EPS-nonproducing cells provide a further basis for understanding the molecular mechanism underlying the production of mannose-rich and high-molecular weight-EPS, as well as clues for artificially increasing the amounts of EPSs and lipids produced.

## INTRODUCTION

The worldwide production of biodiesel-derived crude glycerol increased from 167 kt in 2003 to 2,000 kt in 2012 (1). This product includes glycerol, water, methanol, soap, ash, and matter organic nonglycerol, which are impurities other than water and ash, whose composition depends on the biodiesel source (1). Through a fat-splitting process, the oleochemical industry also generates a methanol-free byproduct, which we refer to as “oleochemical industrial waste,” which has a relatively low concentration of glycerol (8%–20%) (2).

The utilization of purified glycerol and biodiesel-derived crude glycerol by microorganisms is of considerable interest, and many studies have examined the microbiological production of value-added chemicals, including lipids, 1,3-propanediol, 2,3-butanediol, erythritol, arabitol, mannitol, dihydroxyacetone, lactic acid, oxalic acid, succinic acid, malic acid, and citric acid (1, 3–5). In contrast to the many efforts to utilize biodiesel-derived crude glycerol, studies of oleochemical industrial-waste crude glycerol are limited to the production of lipids using oleaginous yeasts reported by Keita et al. and biosurfactants (sophorolipids) using the yeast *Starmerella bombicola* ATCC 22214 reported by Wadekar et al.*,* where Keita et al. and Wadekar et al. referred to the waste as sweetwater (2, 6).

Microorganisms, including bacteria, fungi, and microalgae, can produce extracellular polysaccharides (EPSs), which are released into the surrounding environment (7, 8). Compared with those produced by bacterial cells, EPSs produced by yeasts have the advantages of being low cost, nontoxic, eco-friendly, and easy to extract from culture media and “have received growing attention due to their various applications in the food, cosmetics, and pharmaceutical industries” (8). Several yeasts can produce EPSs from carbon sources, including glucose, sucrose, and starch (8), but only *Candida famata* and *Candida guilliermondii* have been shown to produce EPSs from glycerol (9). EPS production from oleochemical industrial waste crude glycerol has not been reported.

*Rhodotorula toruloides* is a nonconventional, oleaginous yeast that is well known for its industrial potential as a lipid producer due to its high lipid content of more than 70% under certain conditions and its ability to produce carotenoids and enzymes (10, 11). *R. toruloides* utilizes a wide range of substrates and is tolerant to high osmotic stress, low pH, high temperature, and oxidative stress (11). The *R. toruloides* NBRC 8766 strain (synonyms: DSM 4444, NRRL Y-6987, and PYCC 5615) has been used to produce lipids from glycerol (2, 12) and as a target strain for adaptive laboratory evolution to a multistress-tolerant strain under methanol and high glycerol selective pressure (13). Lipid accumulation by this NRRL Y-6987 (synonym NBRC 8766) strain is significantly increased by oleochemical industrial waste, as reported by Keita et al. (2). Recently, Hamacek et al. reported that *R. toruloides* CCT 0783 (synonym NBRC 10076) produces EPS consisting of glucose as a major component from glucose and that “inorganic, but not organic, nitrogen metabolism was associated with the acidification of the culture environment and simultaneous EPS production” (14).

We undertook this research to explore the utilization of oleochemical industrial waste using the *R. toruloides* NBRC 8766 strain.

## MATERIALS AND METHODS

### Media

Oleochemical industrial waste was adjusted to pH 4.8, as was the pure glycerol solution, by the addition of NaOH and autoclaved for 20 min without filtration before or after autoclaving. OW_9.1_YNB medium consisted of 0.67% (wt/vol) yeast nitrogen base without amino acids (YNB) (Becton, Dickinson and Company) and oleochemical industrial waste, yielding a final glycerol content of 9.1% (wt/vol). Gly_9.1_YNB and Glc_9.1_YNB media contained YNB plus 9.1% (wt/vol) pure glycerol and 9.1% (wt/vol) glucose, respectively. Gly_9.1_S1.3Ay medium is the synthetic medium for lipid production (12) (Table S1), but the pH was not adjusted in this study. The compositions of the other media are described in Table 1 and Table S1. YPD medium (pH 5.6) contained 1% (wt/vol) yeast extract (Nacalai Tesque), 2% (wt/vol) tryptone (Nacalai Tesque), and 2% (wt/vol) glucose. YPD solid medium was solidified with 2.0% (wt/vol) agar.

**TABLE 1:** Effect of nitrogen source on EPS production

Medium^*a*^	Nitrogen source	Concentration of NH_4_^+^	EPS production^*b*^	C:N (mol:mol) [C:N (g:g)]
Gly_9.1_S1.3Ay	1.3 g/L AS 1.0 g/L yeast extract	20 mM	−	~154 [~129]
Gly_9.1_S1.3A	1.3 g/L AS	20 mM	−	~154 [~129]
Gly_9.1_Sy	1.0 g/L yeast extract		−	
Gly_9.1_S2.5A	2.5 g/L AS	38 mM	+	80 [67.2]
Gly_9.1_S5A	5.0 g/L AS	76 mM	+	40 [33.6]
Gly_9.1_S5Ay	5.0 g/L AS 1.0 g/L yeast extract	76 mM	+	~40 [~33.6]
Gly_9.1_S4.1NH_4_Cl	4.1 g/L NH_4_Cl	76 mM	+	40 [33.6]

^*a*^ All media included 9.1% (wt/vol) pure glycerol as a carbon source.

^*b*^ White flocculent material was formed (+) or not formed (−) when the 7-day culture supernatant was mixed with ethanol.

### Strains and cultivation

The *R. toruloides* NBRC 8766 strain was purchased from the Biological Resource Center, NITE (NBRC). *R. toruloides* Y-27012 and Y-1091 strains were obtained from the USDA-ARS Culture Collection (NRRL). Yeast strains were grown in YPD medium and stored at −80°C in the presence of 16.7% (vol/vol) glycerol. The stored yeast strains were pre-grown on YPD solid medium at 30°C, precultured in 5.0 mL of liquid YPD medium in a 50 mL Erlenmeyer flask for 24 h, collected, washed once with sterilized pure water, and suspended in sterilized pure water. The cells in the suspension were inoculated to reach an OD_600_ of 0.10 into 20 mL of liquid medium in a 100 mL Erlenmeyer flask and cultivated for EPS production. Cultivation in liquid medium was conducted aerobically using a reciprocal shaker (PersonalLt-10F; Taitec, Saitama, Japan) at 30°C and 150 strokes per minute (spm).

### Isolation and analysis of EPSs

EPSs were isolated from the culture as described previously (15) with slight modifications. Briefly, the culture was centrifuged at 18,800 × *g* for 2 min, and the supernatant was mixed with 4 vol. of 99.5% ethanol, yielding a white flocculent material. The 2.0 mL culture was centrifuged unless otherwise stated. After the mixture containing the white flocculent material was centrifuged at 9,840 × *g* for 10 min, the resulting pellet was dissolved in 1.0–2.0 mL of pure water, resulting in an EPS solution. The protein concentration of this solution was determined using Bradford reagent (Sigma Aldrich), after which the EPS solution was freeze-dried. The weight of the protein was subtracted from the weight of the freeze-dried EPSs to yield the weight of the EPSs produced. The EPS weight is expressed as mg per mL of culture medium.

To prepare the EPS for GC‒MS analyses and molecular weight determination, the culture was centrifuged at 5,000 × *g* for 2 min and treated as above, except that the EPS solution was deproteinated by the Sevag method (EPS solution:chloroform:*n*-butanol = 5:4:1, vol/vol/vol) (16) and dialyzed against pure water before freeze-drying unless otherwise stated. The sugar components of EPS were analyzed according to the method reported by Matsuzaki et al. (17). In brief, EPS (200 µg) and a *myo*-inositol standard (Wako, 100 µg) were treated with 0.2 mL of 5% hydrogen chloride methanol solution (Nacalai Tesque Inc.) in a thick glass tube in a microwave oven (500 W, 30 s). After cooling, 20 mg of silver carbonate was added to remove chloride ions, and silver chloride and excess silver carbonate were removed by filtration. The filtered liquid was concentrated under an N_2_ stream. Trimethylsilyl (TMS) derivatives of sugars were prepared by adding 0.1 mL Tri-Sil HTP (HDMS:TMCS:Pyridine) Reagent (Thermo Fisher Scientific Inc., Waltham, MA, USA) and incubating at 60°C for 30 min. TMS sugars were analyzed by GC‒MS (7890GC/5977MSD; Agilent Technologies Inc.) with a capillary column (30 m × 0.25 mm internal diameter, 0.25 μm film thickness, DB-5 MS; Agilent Technologies Inc.) maintained at 80°C for 2 min and increased to 180°C at 20°C/min and then to 240°C at 4°C/min. D-mannose (Nacalai), D-galactose (Nacalai), and L-fucose (Sigma Aldrich Corp.) were used as standards.

The molecular weight distributions of EPSs (500 µg) were determined by high-performance size exclusion chromatography with Shodex OHpak SB-807G (Guard), SB-807 HQ, and SB-806M HQ (8.0 mm ID × 300 mm length; Showa Denko KK, Tokyo, Japan) at 35°C and estimated using dextran standards (35, 150, 270, and 670 k from Sigma Aldrich Corp., 3,755 k from American Polymer Standards Corp., Mentor, OH, USA).

### Fluorescence microscopy

Nile red staining was conducted as previously described (18). Fluorescence microscopic observation was performed using an EVOS M7000 with an RFP light cube (Thermo Fisher Scientific).

### TEM analysis

TEM analysis was performed using the rapid freezing and freeze-substitution method (19) at Tokai Electron Microscopy, Inc. (Japan), except that frozen samples were placed in 2% glutaraldehyde and 1% tannic acid in ethanol and 2% distilled water. PATAg staining was conducted as previously described (20). Briefly, the grids were incubated with 1% periodic acid for 20 min, washed with distilled water, incubated with 0.5% thiocarbohydrazide in 20% acetic acid for 60 min, washed with 10% acetic acid and distilled water, incubated with 1% silver proteinate for 60 min, and then washed with distilled water.

### Other analyses

The glucose and glycerol concentrations in the culture supernatants were determined using a Wako glucose C II test (Fujifilm Wako Pure Chemical Corp.) and F-kit glycerol (148270: J.K. International), respectively. The pH of the culture was determined using a LAQUA twin-pH-33 (Horiba, Japan). Unless otherwise stated, the averages and standard deviations (SDs) of the data obtained from three independent experiments are shown. Unpaired *t*-test was conducted with a two-sided Welch’s *t* test. Repeated measure ANOVA was also used for analyzing longitudinal data. The content of cellular lipids was determined by the sulfo-phospho-vanillin method as previously described (21). Briefly, the collected cells were washed twice with pure water, freeze-dried, and dissolved in 18 M H_2_SO_4_ to reach 2.5 mg/mL. A portion of the solution (up to 20 µL) was heated at 100°C for 10 min in 200 µL of 18 M H_2_SO_4_, followed by mixing with 500 µL of phosphor-vanillin reagent (1.2 µg of vanillin per 1.0 mL of 68%, vol/vol, phosphoric acid) and incubating at 37°C for 15 min. After keeping for 45 min in the dark, the *A*_530_ was measured. Oleic acid (FUJIFILM Wako) was used as a standard.

## RESULTS

### Production of EPS from glycerol by *R. toruloides* strains

**Fig 1:**
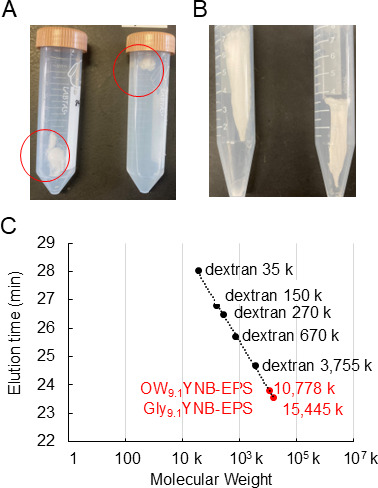
EPS production by the *R. toruloides* NBRC 8766 strain from oleochemical industrial waste crude glycerol and pure glycerol. (**A**) White flocculent materials (circled in red) from OW_9.1_YNB (left) and Gly_9.1_YNB (right). The supernatants of the OW_9.1_YNB-derived culture (6.7 mL) and the Gly_9.1_YNB-derived culture (8.9 mL) were mixed with 4 vol. of 99.5% ethanol. (**B**) Dried white materials from OW_9.1_YNB (left) and Gly_9.1_YNB (right). (**C**) Molecular weight determination of OW_9.1_YNB-EPS and Gly_9.1_YNB-EPS.

To explore the utilization of oleochemical industrial waste, we cultivated the *R. toruloides* NBRC 8766 strain in liquid OW_9.1_YNB medium (YNB plus oleochemical industrial waste (including a final concentration of 9.1% [wt/vol] glycerol) and analyzed the lipids in the cells. During the examination of the utilization, we unexpectedly observed a slight increase in medium viscosity after 5 days and assumed that this viscous medium might contain a high-molecular-weight (HMW) material, such as an EPS. To confirm this, we cultured the *R. toruloides* NBRC 8766 strain in liquid OW_9.1_YNB and Gly_9.1_YNB (YNB plus 9.1% [wt/vol] pure glycerol) media and isolated the HMW material. The supernatants of 5 days growth cultures when mixed with 4 vol. of 99.5% ethanol resulted in the formation of white flocculent materials for both media (Fig. 1A). These materials were collected and freeze-dried, resulting in the formation of dried white materials (Fig. 1B).

After the materials were dissolved in pure water and deproteinated by the Sevag method, the molecular weights of the materials from OW_9.1_YNB and Gly_9.1_YNB media were determined to be >3,755 k (dextran standard) and estimated to be 10,778 kDa and 15,445 kDa, respectively (Fig. 1C). The purified materials were further dialyzed against pure water. GC‒MS analysis of the materials revealed that the material from OW_9.1_YNB medium consisted of (mol %) mannose (97.4), galactose (1.6), and fucose (1.0) residues, whereas the material from Gly_9.1_YNB medium consisted of (mol %) mannose (92.8), galactose (3.5), and fucose (3.7) residues (Fig. S1). *N*-acetylated carbohydrates were not detected. Thus, we concluded that the materials were mannose-rich and HMW-EPSs and called them OW_9.1_YNB-EPS and Gly_9.1_YNB-EPS.

We next examined whether other *R. toruloides* strains could produce EPSs. *R. toruloides* NRRL Y-27012 and NRRL Y-1091 strains, which produce lipids from glycerol (22) or from lignocellulose-derived sugars (23), were cultivated in liquid Gly_9.1_YNB. Like those of the NBRC 8766 strain, white flocculent materials formed (Fig. S2), suggesting that the *R. toruloides* NRRL Y-27012 and NRRL Y-1091 strains also produce EPS.

### Enhanced growth, glycerol consumption, and EPS production by oleochemical industrial waste components

The *R. toruloides* NBRC 8766 and NRRL Y-27012 strains were cultured in liquid OW_9.1_YNB, Gly_9.1_YNB, or Glc_9.1_YNB (YNB plus 9.1% [wt/vol] glucose) medium. The growth and the concentration of glycerol or glucose in the culture supernatant, as well as the weight of EPS, were measured after 3, 5, and 7 days of cultivation. The white flocculent material produced by *R. toruloides* NRRL Y-27012 was regarded as EPS. Small amounts of protein (0.00%–0.71%, wt/wt) were included in the dried white flocculent materials.

**Fig 2:**
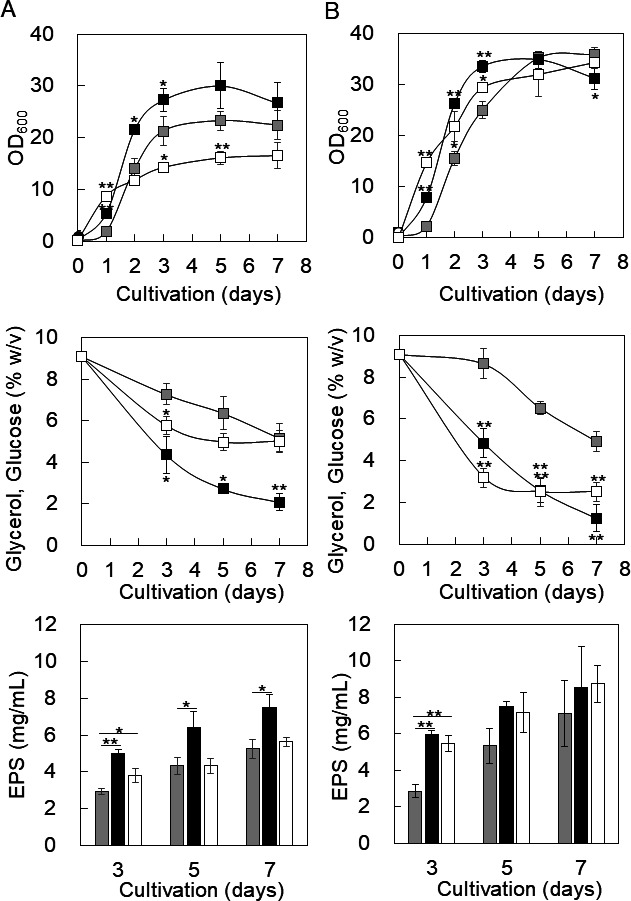
Effects of compounds in oleochemical industrial waste. Effects of compounds in oleochemical industrial waste on the growth (upper panels), consumption of glycerol or glucose in the supernatants (middle panels), and EPS production (bottom panels) of *R. toruloides* NBRC 8766 (**A**) and NRRL Y-27012 (**B**) strains. The strains were cultivated in Gly_9.1_YNB medium (gray), OW_9.1_YNB (black), or Glc_9.1_YNB (white). *P < 0.05, **P < 0.01 vs data obtained from the Gly_9.1_YNB medium.

Regarding the comparison of data from the Gly_9.1_YNB and OW_9.1_YNB media for both strains (Fig. 2), the growth and EPS production were greater in the OW_9.1_YNB medium than in the Gly_9.1_YNB medium. The residual glycerol concentrations in the OW_9.1_YNB culture were also lower than in the Gly_9.1_YNB culture. Several statistically significant differences were observed (Fig. 2). Repeated measure ANOVA revealed statistically significant differences, but no interactions except for an interaction between media and days for the NRRL Y-27012 strain growth (Tables S2 and S3). These data indicate that components of oleochemical industrial waste can increase growth, glycerol consumption, and EPS production, which suggests that oleochemical industrial waste is a suitable resource for EPS production.

Regarding the comparison of data from the Gly_9.1_YNB and Glc_9.1_YNB media, the NBRC 8766 strain exhibited lower growth in the Glc_9.1_YNB medium except at 1 day of cultivation, whereas the NRRL Y-27012 strain exhibited higher growth in the Glc_9.1_YNB medium (Fig. 2). Several statistically significant differences were observed (Fig. 2). The residual glucose concentrations in the Glc_9.1_YNB culture of the NBRC 8766 strain were not statistically lower except at 3 days of cultivation, but those were lower in the NRRL Y-27012 strain (Fig. 2). The amounts of EPS produced from the Glc_9.1_YNB medium were statistically higher than those from the Gly_9.1_YNB medium only after 3 days of cultivation for both strains (Fig. 2). Repeated measure ANOVA revealed statistically significant differences, except for EPS production by NBRC 8766 strain, and interactions were also exhibited (Tables S2 and S3). Thus, the effects of glucose on the NBRC 8766 strain differed from those on the NRRL Y-27012 strain (Fig. 2).

### Key factors responsible for EPS production

The *R. toruloides* NBRC 8766 strain (synonyms: DSM 4444 and NRRL Y-6987) was used to produce lipids from glycerol, where EPS production was not described (2, 12). To verify whether EPS is produced when cultivated in the medium described in the literature (12), we cultivated the *R. toruloides* NBRC 8766 strain in a liquid synthetic medium for lipid production (12) but containing 9.1% pure glycerol (Gly_9.1_S1.3Ay medium, Table 1 and Table S1) as well as in a Gly_9.1_YNB medium for up to 7 days. The strain grew better in the Gly_9.1_S1.3Ay medium than in the Gly_9.1_YNB medium (Fig. S3A). When the culture supernatants from the 3-, 5-, and 7-day cultures were mixed with 4 vol. of 99.5% ethanol, the culture supernatants from the 5- and 7-day cultures in the Gly_9.1_YNB medium clearly contained white flocculent materials, i.e., EPS, but any white flocculent materials were not observed in the mixtures from the Gly_9.1_S1.3Ay cultures (Fig. S3A), suggesting that some components present in the Gly_9.1_YNB medium but absent in the Gly_9.1_S1.3Ay medium are key factors responsible for EPS production.

**Fig 3:**
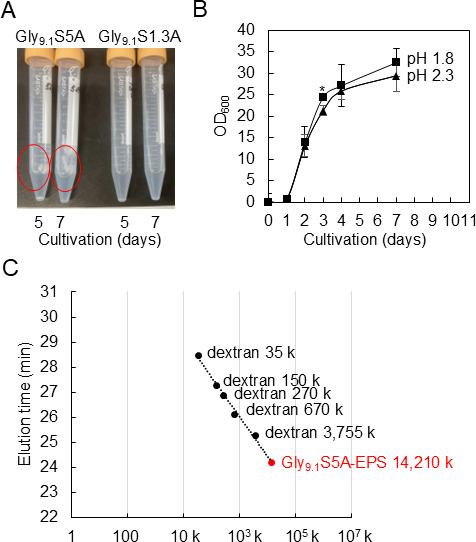
EPS production by the NBRC 8766 strain in Gly_9.1_S5A medium. (**A**) The results of mixing 4 vol. of 99.5% ethanol with the culture supernatants cultivated for the indicated days in the indicated medium. White flocculent materials are circled in red. (**B**) Growth of the NBRC 8766 strain in Gly_9.1_S1.3A (closed triangles) and Gly_9.1_S5A (closed squares) media. *P = 0.0164 < 0.05. The pH values of the cultures after 7 days of cultivation are shown (averages ± SDs of three independent cultivations). (**C**) Molecular weight determination of Gly_9.1_S5A-EPS.

To identify this factor, we compared the composition of the Gly_9.1_YNB medium with that of the Gly_9.1_S1.3Ay medium (Table S1). Independent of a recent publication (14), we initially focused on a nitrogen source, where Gly_9.1_YNB medium contains 5.0 g/L ammonium sulfate (AS) (76 mM NH_4_^+^) and Gly_9.1_S1.3Ay medium includes 1.3 g/L AS (20 mM NH_4_^+^) plus 1.0 g/L yeast extract as a nitrogen source (Table 1 and Table S1). Thus, we cultivated the NBRC 8766 strain for 7 days in modified Gly_9.1_S1.3Ay medium containing various nitrogen sources and tested the EPS production, i.e., whether white flocculent material was formed after the supernatant was mixed with ethanol. As shown in Table 1 and Fig. 3A, white flocculent material formed in the presence of 2.5 g/L AS (38 mM NH_4_^+^), 5.0 g/L AS (76 mM NH_4_^+^), and 4.1 g/L NH_4_Cl (76 mM NH_4_^+^), but not in the presence of 1.3 g/L AS (20 mM NH_4_^+^), or 1.0 g/L yeast extract. The growth of the NBRC 8766 strain in the Gly_9.1_S5A and Gly_9.1_S1.3A media containing 5.0 g/L AS and 1.3 g/L AS, respectively, is shown in Fig. 3B. The strain tended to grow slightly better in Gly_9.1_S5A than in Gly_9.1_S1.3A medium although the difference was statistically significant only at 3 days of cultivation (Fig. 3B). Repeated measure ANOVA also revealed no between-subjects (growth in the two media) variability (*P* = 0.26) or no interaction (*P* = 0.68). The pH values of the cultures at 7 days of culture in Gly_9.1_S5A and Gly_9.1_S1.3A media were 1.80 ± 0.15 and 2.34 ± 0.02 (average ± SDs, *N* = 3, *P* = 0.024 < 0.05), respectively (Fig. 3B).

The dried white flocculate material from the 7-day culture of the Gly_9.1_S5A medium was deproteinated, dialyzed, and analyzed. The molecular weight was estimated to be 14,210 k (Fig. 3C). GC‒MS analysis revealed that the material from the Gly_9.1_S5A medium consisted of (mol %) mannose (96.4), galactose (2.1), and fucose (1.5) residues (Fig. S4). Thus, the material was again identified as mannose-rich and HMW-EPS. The NBRC 8766 strain was also regarded to produce EPS in the presence of 2.5 g/L AS (38 mM NH_4_^+^) due to the formation of white flocculent material (Table 1).

In order to investigate whether the concentration of AS (NH_4_^+^) itself or the resulting acidification of the culture medium is the critical factor for EPS production, effects of the concentration of AS (NH_4_^+^) and the addition of buffer were investigated. The addition of a sodium phosphate buffer (SPB) at a concentration of 50 mM in the presence of 5.0 g/L and 2.5 g/L AS (equivalent to 76 mM and 38 mM of NH_4_^+^, respectively) suppressed the acidification of the culture medium and the production of EPS by the NBRC 8766 strain at 7 days of cultivation (Table S4, Fig. S5A). A higher concentration of AS (NH_4_^+^) resulted in a greater capacity to acidify the medium (Table S4, Fig. S5A). These results suggested that a sufficient concentration of AS (NH_4_^+^) to acidify the medium to a pH of 2.0 or lower (38 mM NH_4_^+^ or more) is key to EPS production. To verify this, we cultivated the NBRC 8766 strain in a Gly_9.1_S1.3A medium containing 1.3 g/L AS (20 mM NH₄^+^) for 7 days in two flasks. We then confirmed that there was no EPS formation. We then artificially acidified one of the flasks to a pH of 1.9–2.0 by adding 1.8 M H₂SO₄. We then cultivated the medium in both flasks for an additional seven days (14 days in total). We observed that EPS was formed in artificially acidified cultures, but not in cultures without acidification (Table S5, Fig. S5B). These data confirmed that a sufficient concentration of AS (38 mM NH₄^+^ or more) to acidify the medium to a pH of 2.0 or lower is key to EPS production under these conditions.

### Microscopic observations

After the NBRC 8766 strain was grown in Gly_9.1_S5A and Gly_9.1_S1.3A media for 5 days, we confirmed that white flocculent material formed in the case of the culture supernatant of the Gly_9.1_S5A medium, but not in the case of that of Gly_9.1_S1.3A medium (Fig. S6); therefore, we called the former cells grown in the Gly_9.1_S5A medium “EPS-producing cells” and the latter cells grown in the Gly_9.1_S1.3A medium “EPS-nonproducing cells” because white flocculent material did not form. The cells were subjected to fluorescence microscopy and transmission electron microscopy (TEM) analyses.

**Fig 4:**
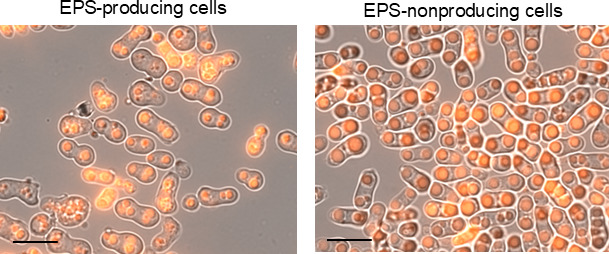
Fluorescence microscopic observations. Fluorescence microscopic observations of the indicated cells of the NBRC 8766 strain cultivated for 5 days and stained with Nile red. The bar represents 10 µm.

For fluorescence microscopy, the cells were stained with Nile red, an excellent probe for the selective staining of intracellular lipid droplets (LDs), which are the predominant fat storage organelles in yeast, plants, and mammals (18). A majority (70%, 44/63 cells in Fig. 4) of the EPS-nonproducing cells showed two clear LDs per cell (Fig. 4), which is typical of *R. toruloides* grown in a medium with high C/N (g/g) ratios (60–90) (24). In contrast, although the EPS-producing cells exhibited LDs, the LDs were smaller and less clear than those in the EPS-nonproducing cells, and a small portion of the cells (12%, 4/34 cells in Fig. 4) had two LDs per cell, whereas a majority (79%, 27/34 cells) had three or more LDs, and some (9%) contained one LD per cell (Fig. 4). Accordingly, the lipid content (0.85 ± 0.09 mg/mg cell dry weight) of the EPS-producing cells was lower than that (1.19 ± 0.01 mg/mg cell dry weight) of the EPS-nonproducing cells (*P* = 0.021, *N* = 3). To obtain more insights, the NBRC 8766 strain was again cultured in Gly_9.1_S5A and Gly_9.1_S1.3A media and observed at 2, 3, 4, and 7 days after staining with Nile red. As shown in Fig. S7, for cells grown in Gly_9.1_S1.3A medium, only the cells at 2 days possessed smaller and less clear LDs, which seemed immature; after 3 days, most of the cells clearly showed two LDs. However, the LDs in the cells grown in the Gly_9.1_S5A medium were smaller and less clear at any phase, suggesting that these LDs were immature at any phase (Fig. S7).

**Fig 5:**
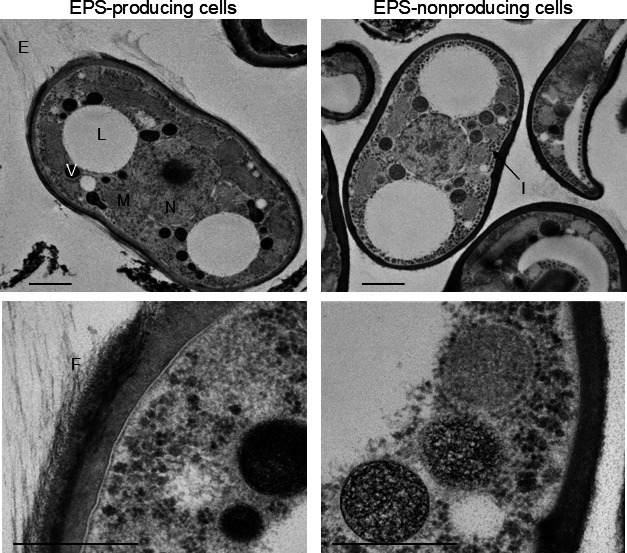
TEM observations of the indicated cells of the NBRC 8766 strain cultivated for 5 days. N, nucleus. M, mitochondria. V, vacuole. L, LD. E, supposed EPS. I, Intracellular granule-like structure. F, fuzz-like structure. Magnification: ×10,600 (upper panels), ×63,300 (bottom panels). Bar: 1.0 µm (upper panels) and 0.50 µm (bottom panels).

**Fig 6:**
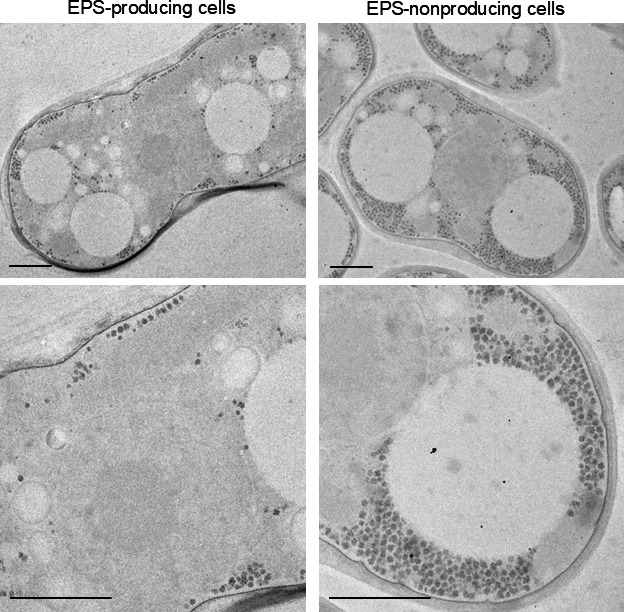
PATAg staining of the indicated cells of the NBRC 8766 strain cultivated for 5 days. Magnification: ×10,600 (upper panels), ×25,300 (bottom panels). Bar: 1.0 µm (upper and bottom panels).

For TEM analysis, the same 5-day-cultured cells shown in Fig. 4 were quickly sandwiched between copper disks, frozen in liquid propane at −175°C, and treated and observed as described in the Materials and Methods. The EPS-producing cells showed marked two-layered cell walls, where the outer layer was darker and qualitatively looked like it was wearing “fuzz,” which is highlighted when enlarged (Fig. 5; Fig. S8). The fuzz-like structure (Fig. 5; Fig. S8F) was assumed to be the EPS released from the cells. Some EPS-producing cells exhibited unidentified dark materials between the cell membrane and cell walls (Fig. S8, “U”). The EPS-nonproducing cells had a one-layered cell wall, and the marked fuzz-like structure was not observed (Fig. 5; Fig. S8). TEM analysis also revealed that the cells, especially the EPS-nonproducing cells, contained intracellular polysaccharide (IPS)-like granules (Fig. 5, I; Fig. S8). To obtain more information, PATAg staining, which detects polysaccharide (25), was performed. The granules, cell membranes, and some cell walls were stained, suggesting that these granules are polysaccharides, i.e., IPS granules, and the granules were richer in EPS-nonproducing cells than in EPS-producing cells (Fig. 6; Fig. S9). Moreover, the size of the granules in the EPS-nonproducing cells (∼63.4 nm in diameter) was greater than that of the EPS-producing cells (∼48.5 nm in diameter).

## DISCUSSION

Previous reports of EPS production by *Rhodotorula* yeasts are summarized in Table 2. In addition to *Rhodotorula* yeasts, a variety of yeasts can produce EPSs (8); however, EPS production from glycerol is limited. Only *C. famata* and *C. guilliermondii* have been shown to produce 1.20 and 1.31 g/L EPS from glycerol (9), respectively, which are low levels compared with other results (Table 2). In this study, we report that *R. toruloides* NBRC 8766 produced mannose-rich and HMW-EPSs from oleochemical industrial waste crude glycerol as well as from pure glycerol. Our results provide the first evidence of EPS production from oleochemical industrial waste crude glycerol by yeast. Compared with the other EPSs produced by *Rhodotorula* yeasts, the mannose-rich and HMW-EPSs had the highest molecular weights (15,445 k, 14,210 k, and 10,778 k), where the EPS produced by *R. mucilaginosa* CICC33013 had the second highest molecular weight, 7,125 k (Table 2). The higher-mannose content is also a property of the mannose-rich and HMW-EPS, where EPSs produced by *R. glutinis* KCTC 7989, *R. babjevae* ATCC 90942, and *R. acheniorum* MC contained 94.4%, 84%, and 69.1%, respectively; however, mannose was not generally the major component in the other EPSs (Table 2).

**TABLE 2:** EPS production by *Rhodotorula* yeasts

Strain^*a*^	Carbon source (g/L)^*b*^	Nitrogen source (g/L)^*c*^	Amounts of EPS produced (g/L)^*d*^	Sugar composition^*e*^ (mol %)	Molecular weight	Ref.
*R. toruloides* NBRC 8766	gly (91)	AS (5) in YNB	5.26	man (92.8), gal (3.5), fuc (3.7)	15,445 k	This study
*R. toruloides* NBRC 8766	gly (91) in OW1	AS (5) in YNB	7.48	man (97.4), gal (1.6), fuc (1.0)	10,778 k	This study
*R. toruloides* NBRC 8766	gly (91)	AS (5) in Gly_9.1_5A	7.3	man (96.4), gal (2.1), fuc (1.5)	14,210 k	This study
*R. toruloides* CCT 0783	glu (120)	AS (5)	5.84	glu (88.83), gal (5.50), man (4.80), xyl, ara (0.87)	NR^*f*^	(14)
*R. mucilaginosa* YL-1	glu (10)	ye (10)	15.1	fuc (1.9), man (27.6), glu (17.6), gal (52.9)	2,960 k 65.7 k	(26)
*R. mucilaginosa* sp. GUMS16	suc (12.5)	AS (50)	28.5	glu (85), man (15)	84 k	(27)
*R. mucilaginosa* CICC 33013	glu (20)	pep (20) ye (10)	6.20	gal (63.1), ara (0.2), glu (18.3), man (18.3)	7,125 k	(28)
*Rhodotorula* sp. strain CAH2	suc (50)	ye (10)	7.48	glu, man, gal	NR	(29)
*R. babjevae* ATCC 90942	glu (30)	AS (2.5) ye (3)	1.6	man (84), glu (16)	1,020 k 500 k 200 k	(30)
*R. minuta* ATCC 10658	sta (15)	defatted soy bean powder (30)	13.8	man (15.4), glu (84.6)	NR	(15)
*R. glutinis*	suc	AS (100)	31.8	glu > gal	118 k	(31)
*R. glutinis* KCTC 7989	glu (30)	AS (20)	4.0	man (94.4), fuc (2.8), glu (1.4), gal (1.4)	100–380 k	(32)
*R. bacarum*	glu (80)	AS (0.6) Soybean cake powder hydrolysate (20)	54	glu (100)	NR	(33)
*R. acheniorum* MC	suc	AS (2.5) ye (1.0)	6.15	ara (3.07), fuc (5.47), man (69.13), gal (8.30), glu (13.73)	NR	(34)

^*a*^ *R*., *Rhodotorula.*

^*b*^ gly, glycerol, glu, glucose, suc, sucrose, sta, starch.

^*c*^ AS, ammonium sulfate, ye, yeast extract, pep, peptone.

^*d*^ Maximum amounts of EPS produced.

^*e*^ man, mannose, gal, galactose, fuc, fucose, xyl, xylose, ara, arabinose.

^*f*^ NR, not reported.

Keita et al. reported pioneering work to explore the utilization of oleochemical industrial waste to produce lipids using oleaginous yeasts and reported that oleochemical industrial waste increased lipid accumulation in several strains, especially *R. toruloides* NRRL Y-6987 (synonym NBRC 8766) (2). We observed a similar increase in EPS production in this study (Fig. 2). Thus, these enhancing effects of industrial oleochemical waste appear to be common regardless of its source. Thus, this study indicates that oleochemical industrial waste is a cost-effective and promising feedstock for the microbial production of not only lipids, carotenoids, and enzymes but also EPS and that the *R. toruloides* NBRC 8766 strain is a promising producer of lipids and EPS.

One of our striking findings is the ammonium ion (NH_4_^+^)-concentration-dependent EPS production by the *R. toruloides* NBRC 8766 strain from glycerol. The media used for lipid production from glycerol using the *R. toruloides* NBRC 8766 strain (synonyms: DSM 4444 and NRRL Y-6987), containing 1.3 g/L AS (12) or less than 0.72 g/L AS (2), were demonstrated to be suitable for lipid production, not EPS production due to the low AS concentration. Hamacek et al. reported EPS production by *R. toruloides* CCT 0783 in a medium containing 5.0 g/L AS (76 mM NH_4_^+^) and 20–120 g/L glucose (initial pH 4.35–4.38, final pH 1.69–1.93), but not in a buffered medium containing 5.0 g/L AS, 20 g/L glucose, and 30 mM K_2_HPO_4_ (initial pH 6.88, final pH 2.69) (14). Hamacek et al. also reported that “inorganic, but not organic, nitrogen metabolism was associated with the acidification of the culture environment and simultaneous EPS production” (14). In this study, we found that EPS (the white flocculent material) was not produced in a non-buffered Gly_9.1_S1.3A medium containing 91 g/L glycerol and 1.3 g/L of inorganic nitrogen source (AS) (Fig. 3A; Fig. S3B and S5; Table 1; Tables S4 and S5). Our further research into the effects of the concentration of AS (NH_4_^+^) and the addition of SPB (Table S4, Fig. S5A) and, in particular, into the effect of the artificial acidification of the culture medium (Table S4, Fig. S5B) on EPS production clearly demonstrated that a sufficient concentration of AS (38 mM NH_4_^+^ or more) to acidify the medium to a pH of 2.0 or lower is key to EPS production under these conditions. It was critical that we found that EPS was not produced in the Gly_9.1_S1.3A medium because this enabled us to conduct the artificial acidification experiment (Table S4, Fig. S5). It was, therefore, hypothesized that the *R. toruloides* NBRC 8766 strain produces EPS in response to extracellular acidity. In the yeast *Saccharomyces cerevisiae*, “NH_4_^+^ uptake is facilitated by ammonium permeases, which transport the NH_4_^+^ ion, resulting in ATP expenditure to maintain the intracellular charge balance and pH by proton export using the plasma membrane-bound H^+^-ATPase” (35). The *R. toruloides* NBRC 8766 cell may also export protons when assimilating NH_4_^+^. Other *Rhodotorula* yeasts, including *R. toruloides* CCT 0783, have been reported to produce EPS in a medium containing 2.5–20 g/L AS (Table 2) and the final culture pH decreasing to below 2.0 (14, 32) or approximately 2.0 (34). This suggests that these *Rhodotorula* yeasts produce EPS in response to extracellular acidity, as is the case with *R. toruloides* NBRC 8766. However, some *Rhodotorula* yeasts, such as *R. mucilaginosa* YL-1, *R. mucilaginosa* CICC 33013, *Rhodotorula* sp. strain CAH2, and *R. minuta* ATCC 10658, produced significant quantities of EPS (6.2–15.1 g/L EPS) in the absence of NH_4_^+^, using 10 g/L of yeast extract, 10 g/L of yeast extract plus 20 g/L of peptone, 10 g/L of yeast extract, or 30 g/L of defatted soybean powder as nitrogen sources (15, 26, 28, 29). This suggests that the mechanism underlying the EPS production of these *Rhodotorula* yeasts differs from that of *R. toruloides* NBRC 8766.

Moreover, we revealed the differences in morphology between EPS-producing cells grown in a Gly_9.1_S5A medium (in the presence of 5.0 g/L AS [76 mM NH_4_^+^]) and EPS-nonproducing cells grown in a Gly_9.1_S1.3A medium (in the presence of 1.3 g/L AS [20 mM NH_4_^+^]). They apparently differed at least in the size, number per cell, and shape of the LDs (small vs large, generally more than three vs two, and immature LDs vs clear LDs), in the structure of the cell wall (two-layered with fuzz-like structure vs one-layered), and in the number of IPS granules (a few vs many, small [∼48.5 nm] or large [63.4 nm]) (Fig. 4 to 6, S7–S9). These microscopic observations of NBRC 8766 strains provide some clues for artificially increasing the amounts of EPSs and lipids produced, e.g., converting the lipids in the smaller and immature LDs into EPSs in EPS-producing cells or converting the polysaccharides in IPS granules into lipids in the LDs in EPS-nonproducing cells with the help of microbial biotechnology.

Finally, we speculate that the *R. toruloides* NBRC 8766 strain keeps the LDs immature and synthesizes IPSs and secretes them as EPS out of the cell in response to extracellular acidity caused by a sufficient concentration of NH_4_^+^ when grown in a Gly_9.1_S5A medium. The outer and darker cell wall with fuzz-like structures in the two-layered cell would reflect the secreted EPS; the unidentified material between the cell membrane and cell wall might also be the polysaccharides to be secreted. We further speculate that the strain converts glycerol into lipids and polysaccharides and accumulates and holds them in the forms of LDs and IPS granules when grown in a Gly_9.1_S1.3A medium. We suppose that the accumulated IPS granules were possibly released as an EPS following the artificial acidification of the medium (Table S4, Fig. S5).

## Data Availability

The data sets used and/or analyzed during the current study are available from the corresponding author upon reasonable request.

## SUPPLEMENTAL MATERIAL

Supplemental figures and tables spectrum.00429-26-s0001.docx Fig. S1 to S9 and Tables S1 to S5.

ASM does not own the copyrights to Supplemental Material that may be linked to, or accessed through, an article. The authors have granted ASM a non-exclusive, world-wide license to publish the Supplemental Material files. Please contact the corresponding author directly for reuse.
