# Supplementary material for: Ammonium ion concentration-dependent production of extracellular polysaccharides from glycerol by an oleaginous yeast: visualization of the producer cells
Source: Microbiol Spectr. 2026 Jun 29;14(8):e00429-26. doi: 10.1128/spectrum.00429-26 (PMC13436021; doi:10.1128/spectrum.00429-26)
Supplement: Supplementary file 1 [file spectrum.00429-26-s0001.docx]

**Ammonium ion concentration-dependent production of extracellular polysaccharides from glycerol by an oleaginous yeast: Visualization of the producer cells**

Soyoka Takegawa^1^, Chiaki Matsuzaki^2^, Taiki Hikosaka^3^, Yuji Okabe^3^, Koji Kawai^3^, Shigeyuki Kawai^2^*

^1^ Department of Environmental Science, Faculty of Bioresources and Environmental Sciences, Ishikawa Prefectural University, 1-308 Suematsu, Nonoichi,

Ishikawa 921-8836, Japan

^2^ Laboratory for Environmental Biotechnology, Research Institute for Bioresources and Biotechnology, Ishikawa Prefectural University, 1-308 Suematsu, Nonoichi,

Ishikawa 921-8836, Japan

^3^ Miyoshi Oil & Fat Co., Ltd., Olinas Tower 13th floor, 4-1-3, Taihei, Sumida-ku, Tokyo 130-0012, Japan

^*^ Corresponding author

kawais@ishikawa-pu.ac.jp

Tel: +81-76-227-7518; Fax: +81-76-227-7557

**Supplementary Figures**

**Fig. S1. GC‒MS analysis of OW_9.1_YNB-EPS and Gly_9.1_YNB-EPS.** Authentic sugars: Man, mannose, Gal, galactose, Fuc, and fucose.

**Fig. S2. EPS production by *R. toruloides* NRRL strains.** A. Results of mixing 4 vol. of 99.5% ethanol with the culture supernatants of the indicated strains cultivated for 7 days in Gly_9.1_YNB medium. White flocculent materials are circled in red. 8766, NBRC 8766 strain. B. Weights of EPSs from 5-day cultures in Gly_9.1_YNB medium. The data for the NBRC 8766 strain are the same as those in Fig. 2.

**Fig. S3. Growth and formation of white flocculent materials.** A. Growth of the NBRC 8766 strain in Gly_9.1_YNB (gray squares) and Gly_9.1_S1.3Ay (gray triangles) media. B. Results of mixing 4 vol. of 99.5% ethanol with the culture supernatants cultivated for the indicated days in the indicated medium. White flocculent materials are circled in red.

**Fig. S4. GC‒MS analysis of Gly_9.1_S5A-EPS.** Authentic sugars: Man, mannose, Gal, galactose, Fuc, and fucose. *N*-acetylated carbohydrates were not detected.

**Fig. S5. An AS concentration sufficient to acidify the medium to a pH of 2.0 or lower is key to EPS production.** A. Results of mixing 4 vol. of 99.5% ethanol with the culture supernatants cultivated for 7 days in Gly_9.1_S5A (no. 1 and 4), Gly_9.1_S2.5A (no. 2 and 5), or Gly_9.1_S1.3A (no. 3 and 6) media in the absence or presence of SPB at 50 mM. B. Results of mixing 4 vol. of 99.5% ethanol with the culture supernatants cultivated for 7 and 14 days in Gly_9.1_S1.3A medium. In the case of cultivation for 14 days, the medium was artificially acidified to a pH 1.89 by adding 1.8 M H_2_SO_4_ after 7 days of cultivation in flask no. 718 (artificial acidification, Yes) or not in flask No. 717 (artificial acidification, No), and was then cultivated for a further 7 days (total 14 days). Data from the exp. 7 in Table S5 are shown. AB: White flocculent materials (EPSs) are circled in red.

**Fig. S6. Results of mixing 4 vol. of 99.5% ethanol with the culture supernatants of the cells cultivated in the indicated medium for microscopic observation.** The NBRC 8766 strains were cultivated for 5 days in the indicated medium. White flocculent materials are circled in red.

**Fig. S7. Fluorescence microscopic observations of the indicated cells of the NBRC 8766 strain cultured for the indicated period and stained with Nile red.** Bar: 10 µm.

**Fig. S8.** **TEM observations of the indicated cells of the NBRC 8766 strain cultured for 5 days.** “U”, unidentified dark material between the cell membrane and cell walls. F, fuzz-like structure. Magnification: ×10,600 (A), ×63,300 (B). Bar: 1.0 µm (A) and 0.50 µm (B). Each photo in (A) corresponds to each photo in (B).

**Fig. S9.** **PATAg staining of the indicated cells of the NBRC 8766 strain cultured for 5 days.** Magnification: ×10,600 (A), ×25,300 (B). Bar: 1.0 µm (AB). Each photo in (A) corresponds to each photo in (B). The bar represents 1.0 µm.

**Fig. S1**

**Fig. S2**

**Fig. S3**

**Fig. S4**

**Fig. S5**

**Fig. S6**

**Fig. S7**

**Fig. S8A**

**Fig. S7B**

**Fig. S9A**

**Fig. S9B**

**Supplementary Tables**

**Table S1 Media composition.**

**Table S2 Results of repeated measure ANOVA of the data in Figure 2A.**

**Table S3 Results of repeated measure ANOVA of the data in Figure 2B.**

**Table S4 Effects of the concentration of AS (NH_4_^+^) and sodium phosphate buffer (SPB) on EPS production by *R. toruloides* NBRC 8766 and on the pH of the culture medium after 7 days of cultivation.**

**Table S5 Effect of the artificial acidification of the culture medium on EPS production.**

| Table S1 Media compositions. | | |  |  |
| --- | --- | --- | --- | --- |
|  | Gly_9.1_YNB | Gly_9.1_S1.3Ay | Gly_9.1_S1.3A | Gly_9.1_S5A |
|  | mg | mg | mg | mg |
| Yeast extract |  | 1000 |  |  |
| AS | 5000 | 1300 | 1300 | 5000 |
| NaNO_3_ |  |  |  |  |
| Na_2_HPO_4_ |  | 950 | | |
| KH_2_PO_4_ | 1,000 | 2700 | | |
| MgSO_4_ | 500 | 98 | | |
| CaCl_2_ | 100 | 30 | | |
| NaCl | 100 |  |  |  |
| Citric acid |  | 5.2 | | |
| H_2_SO_4_ |  | 1.84 | | |
| FeSO_4_ |  | 3.01 | | |
| FeCl_3_ | 0.2 |  |  |  |
| CuSO_4_ | 0.04 |  |  |  |
| ZnSO_4_ | 0.4 | 0.56 | | |
| MnSO_4_ | 0.4 | 0.68 | | |
| Na_2_MoO_4_ | 0.2 |  |  |  |
| KI | 0.1 |  |  |  |
| Boric acid | 0.5 |  |  |  |
| Biotin | 0.002 |  |  |  |
| Ca pantothenate | 0.4 |  |  |  |
| Folic acid | 0.002 |  |  |  |
| Inositol | 2 |  |  |  |
| Niacin | 0.4 |  |  |  |
| Pyridoxine, HCl | 0.4 |  |  |  |
| Riboflavin | 0.2 |  |  |  |
| Thiamine, HCl | 0.4 |  |  |  |
| p-Aminobenzoic acid | 0.2 |  |  |  |
| Pure glycerol | 91 g | | | |
| Pure water | To 1,000 mL | | | |

| Table S2 Results of repeated measure ANOVA of the data in Figure 2A. | | | | | | |
| --- | --- | --- | --- | --- | --- | --- |
|  | SS | df | MS | F | p | F (0.95) |
| Growth of NBRC 8766 |  |  |  |  |  |  |
| Media (Gly_9.1_YNB vs OW_9.1_YNB) |  |  |  |  |  |  |
| BSV | 237.4 | 1 | 237.4 | 19.7 | 0.011 | 7.7 |
| WSV (days) | 2128.5 | 4 | 532.1 | 105.2 | 0.000 | 3.0 |
| Interaction, media × days | 17.9 | 4 | 4.5 | 0.9 | 0.495 | 3.0 |
| Media (Gly_9.1_YNB vs Glc_9.1_YNB) |  |  |  |  |  |  |
| BSV | 71.9 | 1 | 71.9 | 8.4 | 0.044 | 7.7 |
| WSV (days) | 882.9 | 4 | 220.7 | 122.9 | 0.000 | 3.0 |
| Interaction, media × days | 207.3 | 4 | 51.8 | 28.8 | 0.000 | 3.0 |
| Consumption of carbons by NBRC 8766 |  |  |  |  |  |  |
| Media (Gly_9.1_YNB vs OW_9.1_YNB) |  |  |  |  |  |  |
| BSV | 46.4 | 1 | 46.4 | 117.4 | 0.000 | 7.7 |
| WSV (days) | 14.5 | 2 | 7.3 | 18.4 | 0.001 | 4.5 |
| Interaction, media × days | 0.4 | 2 | 0.2 | 0.6 | 0.594 | 4.5 |
| Media (Gly_9.1_YNB vs Glc_9.1_YNB) |  |  |  |  |  |  |
| BSV | 4.6 | 1 | 4.6 | 65.5 | 0.001 | 7.7 |
| WSV (days) | 6.2 | 2 | 3.1 | 6.6 | 0.020 | 4.5 |
| Interaction, media × days | 1.7 | 2 | 0.9 | 1.9 | 0.219 | 4.5 |
| EPS production by NBRC 8766 |  |  |  |  |  |  |
| Media (Gly_9.1_YNB vs OW_9.1_YNB) |  |  |  |  |  |  |
| BSV | 81.7 | 1 | 81.7 | 33.3 | 0.004 | 7.7 |
| WSV (days) | 69.5 | 2 | 34.7 | 66.2 | 0.000 | 4.5 |
| Interaction, media × days | 0.1 | 2 | 0.0 | 0.1 | 0.934 | 4.5 |
| Media (Gly_9.1_YNB vs Glc_9.1_YNB) |  |  |  |  |  |  |
| BSV | 3.0 | 1 | 3.0 | 2.8 | 0.167 | 7.7 |
| WSV (days) | 51.9 | 2 | 25.9 | 88.8 | 0.000 | 4.5 |
| Interaction, media × days | 2.3 | 2 | 1.2 | 4.0 | 0.063 | 4.5 |
| SS, sum of squares, MS, mean square, BSV, between-subjects variability, WSV, within-subject variation | | | | | | |

| Table S3 Results of repeated measure ANOVA of the data in Figure 2B. | | | | | | |
| --- | --- | --- | --- | --- | --- | --- |
|  | SS | df | MS | F | p | F (0.95) |
| Growth of NRRL Y-27012 |  |  |  |  |  |  |
| Media (Gly_9.1_YNB vs OW_9.1_YNB) |  |  |  |  |  |  |
| BSV | 119.6 | 1 | 119.6 | 28.1 | 0.006 | 7.7 |
| WSV (days) | 3660.8 | 4 | 915.2 | 799.4 | 0.000 | 3.0 |
| Interaction, media × days | 247.0 | 4 | 61.8 | 53.9 | 0.000 | 3.0 |
| Media (Gly_9.1_YNB vs Glc_9.1_YNB) |  |  |  |  |  |  |
| BSV | 102.3 | 1 | 102.3 | 16.4 | 0.016 | 7.7 |
| WSV (days) | 2991.8 | 4 | 747.9 | 225.7 | 0.000 | 3.0 |
| Interaction, media × days | 242.6 | 4 | 60.6 | 18.3 | 0.000 | 3.0 |
| Consumption of carbons by NRRL Y-27012 |  |  |  |  |  |  |
| Media (Gly_9.1_YNB vs OW_9.1_YNB) |  |  |  |  |  |  |
| BSV | 64.9 | 1 | 64.9 | 87.2 | 0.001 | 7.7 |
| WSV (days) | 41.0 | 2 | 20.5 | 148.3 | 0.000 | 4.5 |
| Interaction, media × days | 0.1 | 2 | 0.0 | 0.2 | 0.824 | 4.5 |
| Media (Gly_9.1_YNB vs Glc_9.1_YNB) |  |  |  |  |  |  |
| BSV | 70.2 | 1 | 70.2 | 214.3 | 0.000 | 7.7 |
| WSV (days) | 14.9 | 2 | 7.5 | 27.4 | 0.000 | 4.5 |
| Interaction, media × days | 7.1 | 2 | 3.5 | 13.0 | 0.003 | 4.5 |
| EPS production by NRRL Y-27012 |  |  |  |  |  |  |
| Media (Gly_9.1_YNB vs OW_9.1_YNB) |  |  |  |  |  |  |
| BSV | 89.5 | 1 | 89.5 | 8.8 | 0.041 | 7.7 |
| WSV (days) | 141.9 | 2 | 70.9 | 16.2 | 0.002 | 4.5 |
| Interaction, media × days | 9.0 | 2 | 4.5 | 1.0 | 0.399 | 4.5 |
| Media (Gly_9.1_YNB vs Glc_9.1_YNB) |  |  |  |  |  |  |
| BSV | 73.8 | 1 | 73.8 | 9.7 | 0.036 | 7.7 |
| WSV (days) | 171.2 | 2 | 85.6 | 36.5 | 0.000 | 4.5 |
| Interaction, media × days | 3.5 | 2 | 1.8 | 0.8 | 0.501 | 4.5 |
| SS, sum of squares, MS, mean square, BSV, between-subjects variability, WSV, within-subject variation | | | | | | |

| Table S4 Effects of the concentration of AS (NH_4_^+^) and sodium phosphate buffer (SPB) on EPS production by *R. toruloides* NBRC 8766 and on the pH of the culture medium after 7 days of cultivation. ^a^ | | | | | | |
| --- | --- | --- | --- | --- | --- | --- |
| Nos. of Exp. | Medium | Initial conc. of AS (g/L) / NH_4_^+^ (mM) | Initial conc. of SPB (mM) | Initial pH of medium ^b^ | EPS production at 7 days ^c^ | pH of the medium at 7 days ^b^ |
| 1 | Gly_9.1_S5A | 5.0 / 76 | 0 | 6.31 ± 0.01 | + | 1.96 ± 0.07 ^e^ |
| 2 | Gly_9.1_S2.5A | 2.5 / 38 | 0 | 6.34 ± 0.01 | + | 1.96 ± 0.09 ^f^ |
| 3 | Gly_9.1_S1.3A | 1.3 / 20 | 0 | 6.60 ± 0.00 | - | 2.34 ± 0.09 |
| 4 | Gly_9.1_S5AB | 5.0 / 76 | 50 | 6.63 ± 0.01 | - ^d^ | 2.37 ± 0.11 |
| 5 | Gly_9.1_S2.5AB | 2.5 / 38 | 50 | 6.67 ± 0.01 | - | 2.94 ± 0.68 |
| 6 | Gly_9.1_S1.3AB | 1.3 / 20 | 50 | 6.31 ± 0.01 | - | 5.40 ± 0.37 |
| ^a^ The experiments were conducted independently three times. The initial OD_600_ was 0.09 in this experiment. SPB (1.0 M, pH 6.4) was used.  ^b^ The averages ± SDs of the three independent experiments are shown.  ^c^ White flocculent material (EPS) was formed (+) or not formed (-) when the 7-day culture supernatant was mixed with ethanol.  A representative result from the three experiments is shown in Fig. S5A.  ^d^ In one of the three experiments, a few thin flocculent materials (approximately 5.0 mm × 0.2 mm) were observed, while no flocculent materials were observed in the other two experiments.  ^e^ Statistically significant differences were observed in the pH of the medium after 7 days compared with that of exp. 3 (p = 0.005) and with that of the exp. 4 (p = 0.009).  ^f^ A statistically significant difference was observed compared with the pH of the 7-day medium of exp. 3 (p = 0.007), but not with that of the exp. 5 (p = 0.129). | | | | | | |

| Table S5 Effect of the artificial acidification of the culture medium on EPS production. ^a^ | | | | | | |
| --- | --- | --- | --- | --- | --- | --- |
| Nos. of Exp. | Nos. of flasks | EPS production at 7 days ^b^ | pH of the medium at 7 days ^b^ | pH after artificial acidification at 7 days | EPS production after additional 7 days (14 days in total) | pH of the medium after additional 7 days (14 days in total) |
| 7 | 717 ^c^ | - | 2.39 ^d^ | na ^e^ | - | 2.35 ^f^ |
|  | 718 | - | 2.38 ^d^ | 1.89 | + | 1.95 ^g^ |
| 8 | 726 ^c^ | - | 2.65 ^d^ | na | - | 2.33 ^f^ |
|  | 727 | - | 2.24 ^d^ | 1.89 | + | 1.75 ^g^ |
| 9 | 735 ^c^ | - | 2.40 ^d^ | na | - | 2.35 ^f^ |
|  | 736 | - | 2.41 ^d^ | 1.97 | + | 1.70 ^g^ |
| ^a^ The experiments were conducted independently three times. The initial OD_600_ was 0.09 in this experiment. The *R. toruloides* NBRC 8766 strain was cultivated in a Gly_9.1_S1.3A medium. A representative result from exp. 7 is shown in Fig. S5B.  ^b^ White flocculent material (EPS) was formed (+) or not formed (-) when the 7-day culture supernatant was mixed with ethanol.  ^c^ They are the three flasks in exp. 3 in Table S4.  ^d^ Average ± SD of the six data was 2.35 ± 0.08.  ^e^ na, Not artificially acidified.  ^f,g^ Average ± SD of the pH of the three data ^f^ was 2.34 ± 0.01, and that of the three data ^g^ was 1.85 ± 0.14. p = 0.016 < 0.05. | | | | | | |
